# Asphyxial Mechanisms in Sand Burial, Findings and Diagnostic Challenges—A Case Report and a Literature Review

**DOI:** 10.3390/diagnostics16111691

**Published:** 2026-05-30

**Authors:** Donato Morena, Anna Claudia Caruso, Martina Padovano, Matteo Scopetti, Vittorio Fineschi

**Affiliations:** 1 Department of Anatomical, Histological, Forensic and Orthopedic Sciences, Sapienza University of Rome, 00161 Rome, Italy; annaclaudia.caruso@uniroma1.it (A.C.C.); vittorio.fineschi@uniroma1.it (V.F.); 2 Forensic Science Police Service, Directorate for the Forensic Science Police and the Cyber Security, Department of Public Security, 00173 Rome, Italy; martina.padovano@uniroma1.it; 3 Department of Medical Surgical Sciences and Translational Medicine, Sapienza University of Rome, 00189 Rome, Italy; matteo.scopetti@uniroma1.it

**Keywords:** sand burial, mechanical asphyxia, thoracic compression, airway obstruction, forensic pathology, confined space fatalities

## Abstract

**Background:** Fatal sand burial is a rare and diagnostically challenging entity in forensic practice. In such cases, death may result from thoracic or thoracoabdominal compression, airway obstruction by particulate material, massive inhalation of sand or soil, or a combination of these mechanisms. External signs may be subtle or absent, making postmortem interpretation highly dependent on a comprehensive, multilevel assessment. **Case Presentation and Methods:** We report the case of a 17-year-old male who died following accidental sand burial caused by the collapse of a self-excavated beach tunnel. External examination, autopsy, histological and toxicological analyses were performed. A review of the literature was also conducted to identify published forensic cases of fatal sand or soil burial and to compare circumstantial, macroscopic, microscopic, and ancillary findings. **Results:** Autopsy revealed marked pulmonary edema and congestion, multivisceral congestion, scattered sand granules within the larynx, and epicardial petechiae. Histological examination demonstrated acute pulmonary emphysema, edema, vascular congestion, and hemorrhagic laterocervical lymph nodes. Overall, the findings were considered most consistent with mechanical asphyxia due to thoracic compression. The literature review identified six eligible studies describing eight fatal cases. Despite the limited sample size and marked heterogeneity, two main diagnostic patterns emerged: compression-related deaths, usually associated with tunnel or beach-hole collapse and minimal or absent particulate material within the airways, and aspiration-/obstruction-related deaths, characterized by abundant or compact sand or soil material within the airways. In cases without massive aspiration, mixed mechanisms may coexist. Pulmonary edema and congestion were the most frequently reported autopsy findings. When available, histological examination appeared useful in distinguishing antemortem from postmortem burial. **Conclusions:** Fatal sand burial should not be regarded as a uniform forensic entity. External examination alone is often insufficient, and accurate diagnosis requires a comprehensive, multidisciplinary approach integrating scene reconstruction, autopsy data, histopathological findings, and ancillary analyses. Hemorrhagic involvement of the laterocervical lymph nodes may represent a potentially relevant but currently underexplored finding, whose diagnostic significance warrants further investigation in analogous cases.

## 1. Introduction

Fatal burial represents a rare cause of asphyxia. Depending on the circumstances, death may result from thoracic or thoracoabdominal compression caused by the weight of overlying sand, from upper airway obstruction and particulate inhalation, or from a combination of both mechanisms [1].

The forensic interpretation of these deaths is often difficult because external signs may be subtle, nonspecific, or even absent. Internal examination may reveal pulmonary edema, pulmonary congestion, emphysematous change, multivisceral congestion, petechial hemorrhages, or airway particulate material, but these findings are variably represented and are not, in isolation, diagnostic of a single mechanism of death [2]. An additional finding that has only rarely been reported is hemorrhagic involvement of the cervical lymph nodes [3].

The main forensic challenge is not merely establishing asphyxia as the cause of death but determining which component of the burial process was primarily lethal: restriction of respiratory movements due to thoracic or thoracoabdominal compression, upper-airway obstruction by particulate material, massive distal aspiration, or a mixed mechanism.

This distinction cannot be based on a single finding. Limited amounts of sand or soil within the mouth, larynx, or proximal airways may reflect agonal breathing, passive contamination during collapse or recovery, or postmortem manipulation, and should not automatically be interpreted as evidence of fatal aspiration. By contrast, compact masses of sand or soil occluding the glottis, trachea, bronchi, or distal airways more strongly support an aspiration- or obstruction-dominant mechanism. In their absence, particularly when the body is recovered from a confined buried position and the scene indicates substantial thoracoabdominal compression, interpretation should favor a compression-dominant mechanism, while still allowing for a contributory mixed component.

From a medico-legal perspective, distinguishing between these mechanisms is important because similar burial scenarios may produce overlapping postmortem findings despite different underlying pathophysiological processes.

In the present study, we report a fatal case of accidental sand burial in a beach tunnel and consider it as a framework to critically reassess the postmortem findings described in comparable forensic cases. Attention is devoted to the differential interpretation of airway particulate material, pulmonary edema and congestion, cardiopulmonary resuscitation (CPR)-related confounders, abdominal injuries, and cervical lymph nodes hemorrhage.

## 2. Case Report

A 17-year-old male died following accidental sand burial at a beach. According to witness accounts, he had dug a sand pit approximately 1.5 m deep and subsequently excavated a tunnel extending inward from the pit. After approximately 45–60 min, the tunnel collapsed, burying him beneath the sand. The victim was not immediately visible, and only after a search of the surrounding area did relatives and bystanders begin excavating the collapse site. The body was eventually retrieved in a prone position, unresponsive and without signs of life. CPR was initiated on site by lifeguards and continued by emergency medical personnel, but resuscitative efforts were unsuccessful.

**Figure 1:**
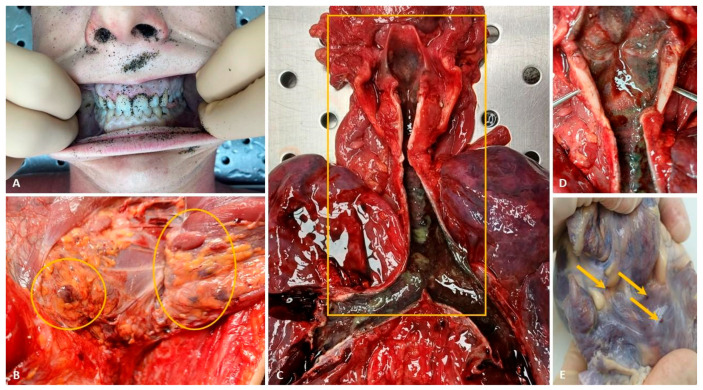
Relevant findings observed during external and gross examination. (**A**) Limited dark granular mineral material consistent with sand was observed involving the nares, lips, and oral cavity. (**B**) Multiple enlarged, firm-elastic laterocervical lymph nodes bilaterally located posterior and lateral to the clavicular heads of the sternocleidomastoid muscles, highlighted by ocher circles. (**C**) En bloc section of the glossopharyngeal–tracheobronchial tract and lungs. The trachea and bronchi showed hyperemic mucosa with abundant reddish and yellowish mucoid material. The lungs exhibited severe congestion and bilateral pulmonary edema. (**D**) Close-up view of the larynx and trachea, showing yellowish mucoid material and scattered sand particles within the laryngeal lumen (**E**) Subcentimetric epicardial petechial hemorrhages (arrows) on the anterior epicardial surface of the pulmonary veins.

At autopsy, the external examination revealed abundant granular dark mineral material consistent with sand involving the hair, eyelids, auricles, nares, lips, oral cavity, and submental region, with diffuse distribution over the remaining body surfaces (Figure 1A).

Bilateral bulbar and palpebral conjunctival hyperemia, as well as subungual cyanosis, were noted. Multiple enlarged, firm-elastic laterocervical lymph nodes were identified bilaterally, posterior and lateral to the clavicular heads of the sternocleidomastoid muscles (Figure 1B). Gross examination revealed marked cerebral and meningeal congestion, with a brain weight of 1516.5 g, together with scattered sand particles within the laryngeal lumen. The trachea and bronchi showed hyperemic mucosa and abundant reddish-yellow mucoid material. The lungs (left: 473.5 g; right: 611 g) exhibited severe congestion and bilateral pulmonary edema (Figure 1C,D). The heart, weighing 301 g, showed epicardial petechial hemorrhages (Figure 1E).

Examination of the abdominal cavity revealed a hepatic laceration (liver weight: 1190 g), associated with approximately 350 mL of blood within the peritoneal recesses and gutters, as well as diffuse multivisceral congestion.

**Figure 2:**
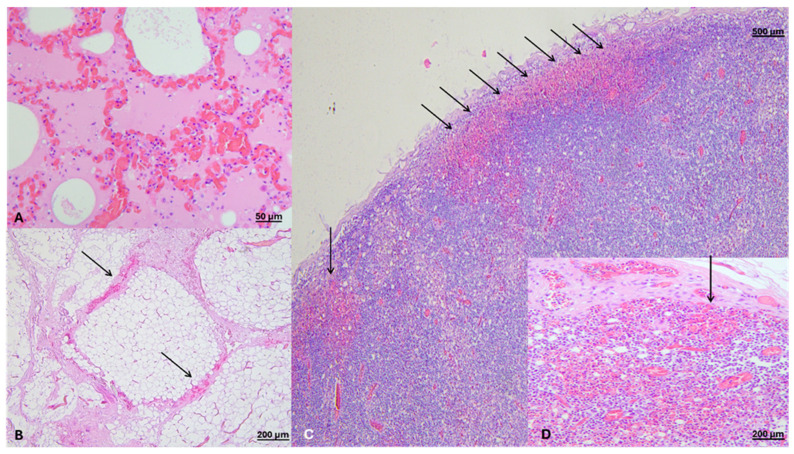
Histological findings of the present case report. (**A**) Lung parenchyma showing intra-alveolar edema, septal congestion, and endoluminal blood stasis (H&E). (**B**) Perinodal fibro-adipose tissue adjacent to the laterocervical lymph nodes, showing focal hemorrhagic infiltration indicated by arrows (H&E). (**C**) Left laterocervical lymph nodes showing preserved architecture withs arrows indicating hemorrhagic extravasation (H&E). (**D**) Higher-power detail of the subcapsular region shown in panel (**C**), with arrows highlighting subcapsular hemorrhagic infiltration and intravascular nodal congestion (H&E).

Histological examination performed with hematoxylin–eosin staining revealed interfascicular edema and terminal hypoxic myocardial changes, including fragmentation of the subpericardial myofibers, interpreted as CPR-related injury. Pulmonary histology demonstrated acute emphysema, interstitial and intra-alveolar edema, marked vascular congestion, focal endobronchial erythrocyte accumulations, focal endobronchial mucoid material, and absence of birefringent particulate matter under polarized light microscopy (Figure 2A). The left laterocervical lymph nodes showed erythrocyte extravasation within the perinodal fibro-adipose tissue and extensive subcapsular hemorrhage (Figure 2B–D).

Splenic tissue also showed focal hemorrhagic findings.

The toxicological examination, performed on central blood and urine samples using broad-spectrum screening for non-volatile organic compounds via gas chromatography–mass spectrometry (GC–MS), yielded negative results for the presence of pharmaceuticals or other exogenous substances of forensic toxicological relevance.

Overall, the findings were considered most consistent with mechanical asphyxia in the setting of accidental sand burial, with thoracic compression regarded as the predominant mechanism rather than massive airway obstruction. This interpretation was supported by the tunnel-collapse scenario, the confined burial position, marked pulmonary and visceral congestion, and the absence of compact sand masses filling or occluding the tracheobronchial tree.

## 3. Materials and Methods

A search for published studies was conducted from inception to November 2025 in PubMed, Scopus, and Google Scholar. The search strategy combined terms related to burial, asphyxia, and forensic postmortem investigation. Search terms included combinations of the following keywords: “bury”, “buried”, “burial”, “fatal”, “forensic”, “autopsy” (the full search strings are reported in Supplementary Materials S1. The nature of the particulate matter was not specified to maximize retrieval sensitivity.

For the eligibility criteria, studies were included if they met the following: (i) reported fatal cases of sand burial; (ii) described asphyxial mechanisms in which sand and/or soil acted as the causal medium, either through thoracic compression impairing respiratory movements, through inhalation leading to airway obstruction, or through a mixed mechanism; (iii) included both external examination and complete autopsy findings; (iv) involved human subjects; and (v) were published from 1990 onwards, to ensure consistency with contemporary forensic autopsy practices.

Studies were excluded if not available in the English language or if they described deaths due to asphyxial or traumatic mechanisms not directly related to sand or soil burial.

Two independent reviewers (D.M. and A.C.C.) conducted the screening and full-text retrieval. Discrepancies during screening and data extraction were resolved through discussion, with arbitration by senior authors (M.S. and V.F.). The reference lists of all retrieved papers were also manually searched by the same two investigators (D.M. and A.C.C.).

A standardized form was used to extract data from the included studies and summarize the results. The extracted information included the author(s), year of publication, age, sex, cause and circumstances of death, findings from the external examination, presence and location of petechiae, upper airway findings, autopsy and histological findings, and any ancillary investigations. Extraction was independently conducted by two reviewers (D.M. and A.C.C.) in duplicate. Senior authors (M.S. and V.F.) were consulted when needed.

The methodological quality of the included case reports was critically appraised using the Joanna Briggs Institute (JBI) Critical Appraisal Checklist for Case Reports [4]. Only this instrument was applied; no additional tools for case series were used, as even studies reporting three or more autopsy cases relevant to this review ultimately contributed to a maximum of two eligible cases.

The JBI checklist for case reports consists of eight structured, criterion-based questions designed to assess the methodological quality and reporting transparency of individual case descriptions. Specifically, the checklist evaluates whether: (Q1) the patient’s demographic characteristics are clearly described; (Q2) the patient’s history is clearly presented and reported as a timeline; (Q3) the current clinical condition at presentation is clearly described; (Q4) diagnostic tests, procedures, and their results are clearly reported; (Q5) the intervention(s) or exposure(s) are clearly described; (Q6) post-intervention or post-exposure clinical condition is clearly described; (Q7) adverse events or unanticipated outcomes are identified and described; and (Q8) the case report provides clear takeaway lessons.

Given the forensic nature of the included material, the appraisal was adapted to prioritize forensic-pathological elements, with clinical aspects considered only when available and relevant. Greater emphasis was placed on the completeness and clarity of autopsy findings, scene and circumstantial reconstruction, toxicological and histological analyses, and the internal consistency of the proposed cause-of-death interpretation, rather than on clinical course or treatment-related variables.

Each included study was independently assessed by three reviewers (D.M., A.C.C., and M.P.). Any disagreements were resolved through discussion or, when necessary, consultation with a senior author (M.S. or V.F.). This structured approach allowed systematic evaluation of methodological rigor and reporting completeness, supporting the assessment of reliability and interpretability of each case for inclusion in the synthesis.

Overall, the review was conducted as a review with systematic search elements. It aimed to map and qualitatively compare published forensic cases of fatal sand or soil burial, focusing on diagnostic patterns rather than quantitative estimates of frequency. No protocol was prospectively registered given that, according to a preliminary search, the available evidence consisted almost exclusively of isolated case reports and small case series. Nevertheless, predefined eligibility criteria, duplicate screening, standardized data extraction, manual reference checking, and transparent reporting of the selection process were applied to ensure methodological rigor.

## 4. Results

The search strategy initially identified 1301 records. After title and abstract screening and removal of 459 duplicates, 842 articles were retained for further evaluation. An additional search conducted through Google Scholar and by screening the reference lists of potentially relevant articles led to the identification of one additional eligible study.

Overall, 12 full-text articles were assessed for eligibility according to the predefined inclusion and exclusion criteria. Ultimately, six studies met the eligibility criteria and were included in the qualitative analysis [1,3,5,6,7,8].

**Figure 3:**
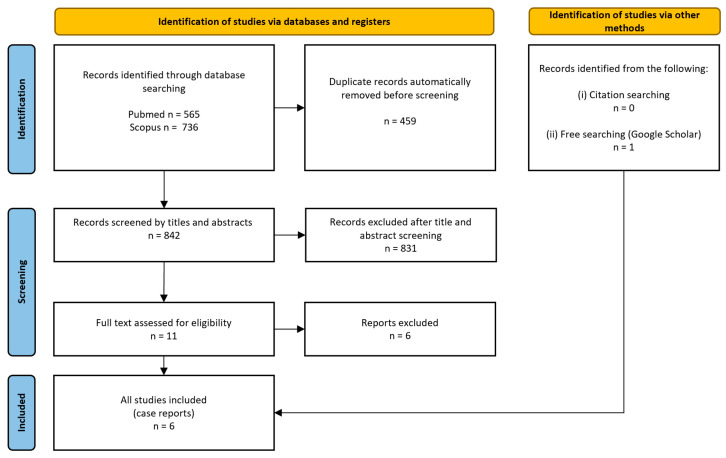
Flow chart of the study selection process.

Figure 3 shows the study selection procedure.

The retrieved studies reported a total of eight individual fatal cases. The main findings of the selected studies are summarized in Table 1.

The included case reports showed generally low risk of bias, particularly in relation to the clarity of diagnostic methods, autopsy findings, scene reconstruction, and forensic interpretation. Most reports provided detailed chronological descriptions and comprehensive postmortem investigations, including radiological imaging, histopathology, toxicological analyses, and photographic documentation.

Nevertheless, some limitations inherent to forensic case reporting were observed. Domains related to clinical intervention and post-intervention outcomes (Q5–Q6) were frequently not applicable, as many cases involved individuals who were deceased at the time of discovery or upon hospital arrival. In addition, demographic and medical background information was sometimes limited, particularly in homicide-related reports where identifying details were intentionally restricted. No included study demonstrated major deficiencies in forensic diagnostic methodology.

Overall, the studies were considered methodologically appropriate for inclusion in the review, with generally robust forensic documentation and low concern for bias affecting the interpretation of asphyxial mechanisms in sand burial deaths. A synthesis of the appraisal is presented in Table 2, while a detailed assessment of individual case reports is provided in Supplementary Materials S2.

Among the reported cases, three resulted from the collapse of self-excavated sand tunnels or beach holes, typically involving children or adolescents during recreational activities [3,7]. Two cases occurred in occupational or construction-related settings, where burial in sand or soil followed collapse of the surrounding material [5]. One case was clearly homicidal in origin [1], one pediatric case occurred in a playground sandbox [6], and another involved the live burial of a severely ill young woman by her partner [8].

Given the small and heterogeneous sample, the cases were not interpreted in terms of numerical predominance of one mechanism over another. Instead, they were categorized according to the diagnostic features supporting compression-dominant, aspiration-/obstruction-dominant, or mixed patterns.

In the recreational tunnel-collapse episodes, the mechanism of death was generally interpreted as mechanical or compressive asphyxia due to thoracic or thoracoabdominal compression in a confined environment. In these cases, external findings were limited or nonspecific and included facial congestion, conjunctival hyperemia or petechiae, cyanosis, and sand contamination of the body surface. Sand within the airways was absent or only minimally represented [3,7], further supporting a predominantly compressive mechanism. At autopsy, the most frequent findings were pulmonary congestion and edema, often associated with generalized visceral congestion and, in some cases, cerebral congestion.

Notably, cervical lymph nodes congestion and hemorrhage were documented in two cases [6], both in association with compressive thoracoabdominal asphyxia; in the same cases, minor hemorrhages of the anterior cervical strap muscles were also described.

By contrast, deaths attributed primarily to aspiration or airway obstruction were more strongly supported when reports described abundant, compact, and anatomically coherent particulate material within the airways.

In one case [5], grayish sand occupied the pharynx and esophagus and filled the larynx, trachea, and bronchi, with radiological evidence of radiopaque material within the aerodigestive tract and histological identification of sand particles in the small bronchioles. In another case [6], obstructive sand masses were removed from the oral cavity and larynx, and massive sand aspiration extended from below the vocal cords to the lobar bronchi. In a further report [1], the oral cavity was filled with soil and sand, the glottis was occluded by a compact soil mass, and the larynx, trachea, and main bronchi contained impacted dark soil and sand; postmortem Computed Tomography (PMCT) also demonstrated radiopaque material filling the upper airways. These cases differed substantially from those in which only sparse or proximal particulate material was identified.

One reported case is diagnostically relevant but should be considered separately from classic massive airway-obstruction patterns [8]. In that case, death was interpreted as mechanical asphyxia due to live burial, mainly related to soil aspiration and airway obstruction, while thoracoabdominal compression was considered less likely because the victim was located close to the ground surface. However, the macroscopic findings did not demonstrate the same degree of gross occlusive airway burden observed in cases characterized by extensive compact particulate material within the airways. Only trace amounts of soil residue were identified macroscopically, although sandy and soil-derived particles were confirmed within the airways and lung tissue by polarized light microscopy. This finding strongly supports antemortem respiration during burial and contributes to the distinction between live burial and postmortem concealment. Nevertheless, it should not be regarded as equivalent to cases characterized by massive macroscopic airway obstruction.

Additional findings reported in individual studies included pulmonary emphysema, gastric sand content, and signs of increased intracranial pressure [5,6,8]. In one case [8], severe natural disease in the form of a perforated duodenal ulcer complicated by peritonitis was considered relevant in explaining the victim’s inability to resist burial.

Histological examinations were available only in a minority of cases. Benroman et al. [5] described sand particles within the small bronchioles, together with alveolar dilatation, hemorrhage, and destruction of the alveolar walls, findings overall consistent with emphysematous change. Kettner et al. [6] reported marked bilateral pulmonary emphysema, with clotted sand masses limited to the lobar bronchi and no aspirated material detected in the smaller airways or alveoli. Byard et al. [1], by contrast, found that histological assessment was severely hampered by putrefactive change, with marked loss of cellular detail. Halasi et al. [8] reported acute emphysema and used polarized light microscopy to detect soil particles in the airways and lung tissue.

Ancillary investigations were reported in five of the eight cases [1,3,5,7,8]. Imaging studies were available in three cases and included two plain radiograph cases [5,7] and one PMCT [1].

One radiograph was normal, with no evidence of a sand bronchogram or trauma [7], whereas the other and the PMCT demonstrated radio-opaque material within the upper airway [1] and aerodigestive tract [5], supporting sand or soil obstruction or aspiration mechanisms. Toxicological analyses were reported in five cases and were consistently negative for alcohol and, when assessed, for drugs [1,3,5,8].

## 5. Discussion

Fatal sand or soil burial is not a single forensic entity but a group of events sharing the same external circumstance and potentially different lethal mechanisms. The central diagnostic issue is the distinction between compression-dominant asphyxia, aspiration- or obstruction-dominant asphyxia, and mixed mechanisms. This distinction cannot be made from one finding alone. It requires integration of scene reconstruction, body position, airway particulate burden, thoracoabdominal constraints, pulmonary and visceral findings, histology, ancillary investigations, and possible confounders such as CPR and recovery maneuvers.

In the limited published literature, the most useful contribution is the identification of diagnostic patterns: abundant or compact particulate material filling or occluding the airways is more consistent with aspiration or obstruction [1,5,6], whereas collapse-related cases with minimal distal airway particulate burden tend to support a compression-based or mixed mechanism [8].

In the present case, the overall circumstantial, macroscopic, and microscopic findings were considered most consistent with mechanical asphyxia in the setting of accidental sand burial, with thoracic compression regarded as the predominant mechanism [3,7]. This interpretation is supported by the collapse of a self-excavated beach tunnel, recovery of the body from a confined buried position, marked pulmonary edema and congestion, diffuse visceral congestion, and the absence of evidence for massive occupation of the tracheobronchial tree by sand. Compression may also partly explain the presence of intraperitoneal blood, likely resulting from hepatic lacerations that were plausibly further worsened during CPR.

Although scattered sand granules were identified in the larynx, the distribution and limited amount of particulate material did not support massive airway obstruction as the primary lethal event. For this reason, a predominantly compression-related mechanism was favored over isolated aspiration, while acknowledging that a minor mixed contribution cannot be excluded with absolute certainty [1,5].

This distinction is of forensic relevance, as the presence of sand in exposed orifices or within the laryngeal lumen does not, per se, imply fatal aspiration. In buried victims, particulate material may enter the mouth, nose, pharynx, or upper larynx during the collapse, agonal respiratory efforts, recovery, or even manipulation of the body. Conversely, a primary lethal mechanism based on airway obstruction is more strongly supported when compact particulate material occludes the glottis, trachea, bronchi, or distal airways in an anatomically coherent distribution that cannot be readily explained by passive contamination alone.

A major medico-legal issue in these deaths is the limited diagnostic value of external examination when considered in isolation. In both the present case and the reviewed literature, external signs were variably represented and often subtle or nonspecific. Findings such as conjunctival hyperemia, cyanosis, facial congestion, and petechiae may support an asphyxial process, but they are neither constant nor pathognomonic [3,6,7].

Their absence cannot exclude mechanical asphyxia, particularly in rapidly evolving compression scenarios or in cases where body position, duration of compression, and terminal events are incompletely reconstructed.

The recent literature has further emphasized the need for cautious interpretation of petechial hemorrhages as indicators of antemortem asphyxia, highlighting their time- and site-dependence and limited sensitivity [9,10]. Accordingly, the relative paucity of external asphyxial stigmata in the present case should not be interpreted as evidence against a compression-related mechanism. Rather, it reinforces the principle that assessment of the mechanism of death in sand burial requires integration of multiple lines of evidence.

Pulmonary edema, pulmonary congestion, and generalized visceral congestion were recurrent autopsy findings across both compression-related and aspiration-/obstruction-related cases [3,5]. Histology, when available, has generally shown edema, vascular congestion, emphysematous change, hemorrhagic damage, or particulate material within the airways [5,6]. In the present case, pulmonary histology demonstrated acute emphysema, interstitial and intra-alveolar edema, and marked vascular congestion, a pattern compatible with acute mechanical asphyxia but not independently diagnostic of the exact mechanism. For this reason, the pulmonary picture must be interpreted in conjunction with the scene dynamics and the amount and distribution of sand within the airways.

From a pathophysiological standpoint, thoracic or thoracoabdominal compression provides a plausible explanatory framework for the principal findings observed in the present case. External compression of the chest wall and diaphragm may severely restrict respiratory movements, impair ventilation, and rapidly lead to hypoxia [11]. In addition, acute compressive forces can induce marked venous hypertension in the cervicofacial region, particularly when occurring during inspiratory efforts against partial airway obstruction, thereby contributing to congestion, cyanosis, petechiae, and related hemorrhagic phenomena [12].

Hemorrhagic changes in the laterocervical lymph nodes were observed in the present case, including erythrocyte extravasation in the perinodal fibro-adipose tissue and subcapsular hemorrhage. Similar findings have been described rarely in fatal sand-burial cases [3]; their significance, however, must be interpreted with substantial caution. Cervical lymph nodes are highly vascularized structures and may show congestion or erythrocyte extravasation in several non-specific settings, including venous stasis, terminal circulatory disturbance, body position, resuscitation-related hemodynamic changes, neck manipulation, and postmortem redistribution. The available literature does not support the use of cervical lymph-node hemorrhage as a specific marker of strangulation, thoracic compression, or any single asphyxial mechanism [13,14,15]. Accordingly, in the present case, laterocervical lymph nodes hemorrhage should be regarded as an ancillary and nonspecific finding and not used as an independent argument in favor of compression-related asphyxia. At most, it may be reported as part of the overall postmortem pattern, provided that alternative explanations are explicitly considered.

Nevertheless, its potential value lies in highlighting the need for systematic cervical sampling in future cases of suspected mechanical asphyxia [16,17,18]. Future studies should define the anatomical level sampled, distinguish capsular, subcapsular, sinusoidal, and perinodal hemorrhage, document CPR and body position, and compare suspected asphyxial deaths with appropriate non-asphyxial controls.

An additional interpretative issue concerns the presence of potential confounding factors. In the present case, CPR maneuvers and traumatic recovery procedures may have contributed to several postmortem findings, including the hepatic laceration and selected myocardial alterations. This distinction is particularly relevant in forensic pathology, as not all hemorrhagic or structural lesions identified at autopsy are necessarily related to the fatal mechanism itself.

CPR may also produce overlapping pulmonary changes, such as edema, vascular congestion, intra-alveolar fluid accumulation, and focal hemorrhagic alterations. Acute lung injury following CPR can mimic or exacerbate agonal and asphyxial findings [19]. Accordingly, the pulmonary edema and congestion observed in this case were interpreted as nonspecific terminal findings, potentially resulting from the combined effects of asphyxia and resuscitative efforts.

Accordingly, the differential diagnosis between accidental and homicidal death must rely on a comprehensive evaluation integrating autopsy findings with crime scene investigation, circumstantial evidence, external signs of restraint or trauma, toxicological evidence of incapacitating substances, and individual vulnerability factors, such as extremes of age or pre-existing pathological conditions that may reduce the victim’s ability to resist a violent assault.

## 6. Limitations

This study has several limitations. The available evidence was mainly limited to case reports, with an extremely small number of eligible cases. In addition, the included reports were highly heterogeneous with respect to scene documentation, autopsy detail, histological sampling, ancillary investigations, and terminology used to describe sand or soil burial. The review was not designed to determine the relative frequency of compression-related versus aspiration/obstruction-related mechanisms. Finally, cervical lymph nodes hemorrhage was not systematically assessed across published reports and therefore cannot be assigned diagnostic specificity. For these reasons, comparisons between compression-related, aspiration-related, and mixed mechanisms should be interpreted as purely descriptive and hypothesis-generating.

## 7. Conclusions

Fatal sand or soil burial is a rare but complex form of mechanical asphyxia. It should not be interpreted as a uniform forensic entity, because death may result from thoracic or thoracoabdominal compression, upper airway obstruction, massive particulate aspiration, or a mixed mechanism. The decisive diagnostic task is therefore to reconstruct the mechanism integrating scene findings, body position, airway particulate distribution, complete autopsy, histology, ancillary investigations, and possible confounders.

In the present case, the overall circumstantial, macroscopic, and microscopic findings were considered most consistent with mechanical asphyxia in the context of accidental sand burial, with thoracic compression regarded as the predominant mechanism over massive airway obstruction. However, as in other forms of fatal mechanical asphyxia, interpretation must remain cautious, especially when findings are subtle and partly non-specific.

The reviewed literature supports the existence of at least two main diagnostic patterns. Compression-related mechanisms are favored in cases involving collapse or confined-space burial, particularly when particulate material is absent, minimal, or restricted to the proximal airways. Aspiration- or obstruction-related mechanisms are more likely when abundant or compact sand or soil fills or occludes the aerodigestive tract, including the glottis, trachea, bronchi, or distal airways, especially when supported by radiological or histological evidence. Mixed mechanisms may be considered in cases where limited airway particulate material coexists with relatively modest compressive forces.

Hemorrhagic changes in laterocervical lymph nodes represent a potentially suggestive but still underexplored finding in this context. However, they should be regarded as nonspecific ancillary features; their main value lies in encouraging standardized documentation and systematic sampling in future cases.

## Figures and Tables

**Table 1:** Summary of macroscopic and microscopic findings in cases of fatal sand burial due to compressive or inhalational mechanisms.

Author	Year	Case	Sex/Age	Circumstances	Case of Death	External Examination	Petechiae (Site)	Upper Airways	Autopsy Findings	Histology	Ancillary Investigations
Zarroug et al. [7]	2004	Case 1	M/10	Collapse of a sand tunnel	Compressive respiratory insufficiency due to thoracic compression	Battle signs and hemotympanum	No	No evidence of trauma or sand debris in the mouth, pharynx, or airway	NR	NR	X-ray: normal findings; no evidence of a sand bronchogram or trauma
Zarroug et al. [7]	2004	Case 2	M/10	Buried in wet sand at a construction site	Compressive respiratory insufficiency due to overwhelming thoracic compression	NR	No	No evidence of trauma or sand debris in the mouth, pharynx, or airway	NR	NR	NR
Benomran et al. [5]	2007	Case report	M/36	Buried under sand discharged from a wheeled loader	Asphyxia due to sand inhalation within the respiratory tract	Sand on clothing and exposed body areas (nostrils, eyes, mouth, auditory canals) and unexposed areas (chest, umbilicus, axillae, pubic region). Small pressure abrasion on the nose	No	Grayish sand in pharynx and esophagus (entire lumen); larynx, trachea, and bronchi filled with the same material	Stomach: small amount of semi-digested food mixed with sand. Lungs: marked congestion and edema; sand particles in bronchioles; frothy edematous fluid expressed on section with moderate compression. Brain and viscera: generalized congestion	Lungs filled with sand particles within small bronchioles; alveolar dilation and hemorrhage with destruction of alveolar walls	X-ray: radiopaque material in pharynx, larynx, trachea, bronchi, and esophagus. Toxicology: no alcohol or drugs detected in blood
Kiryu et al. [3]	2018	Case 1	F/23	Fell into a hole covered with a plastic sheet; suddenly buried by sand	Thoracoabdominal compression by collapsed sand; overwhelming respiratory and diaphragmatic function	Facial congestion; minor bruises of the left lower leg	Petechial hemorrhages of the conjunctivae and oral mucosa; cutaneous petechiae on the neck and upper chest	Minimal sand within the airways	Cervical lymph nodes congestion and hemorrhage; minor hemorrhages in sternohyoid and sternothyroid muscles; marked pulmonary congestion and edema	No	Alcohol not detected in blood or urine
Kiryu et al. [3]	2018	Case 2	M/23	Fell into a hole covered with a plastic sheet; suddenly buried by sand	Thoracoabdominal compression by collapsed sand, overwhelming respiratory and diaphragmatic function	Facial congestion; petechial hemorrhages of the facial skin, conjunctivae, and oral mucosa	Cutaneous petechiae of the neck and upper chest	Sand debris in the oral cavity	Cervical lymph nodes: congestion and hemorrhage. Minor hemorrhages in sternohyoid and sternothyroid muscles. Minimal sand in pharynx, larynx, trachea, and bronchi. Lungs: marked congestion and pulmonary edema	No	Alcohol not detected in blood or urine
Kettner et al. [6]	2008	Case report	M/1–2	Sand ingestion while playing in a sandbox with his older brother	Asphyxia due to airway obstruction	Obstructing sand masses removed from the oral cavity and larynx; sand grains noted only in both groins	No	Below endotracheal intubation: small sand residues on the tongue and laryngeal mucosa. Massive sand aspiration obstructing the airways from 1.5 cm below the vocal cords to the lobar bronchi. Minor, non-obstructive sand in the larynx, oral cavity, and esophagus	Stomach: few sand grains detected. Brain: features of increased intracranial pressure, including effacement of cortical sulci and massive cerebellar tonsillar herniation	Marked bilateral pulmonary emphysema. Clotted sand masses limited to the lobar bronchi; no aspirated material in smaller airways or alveoli	No
Byard et al. [1]	2023	Case report	F/NR	Body found in a shallow sandy grave in a seated position. Hands tied behind the back with a cable tie; blindfolded. Legs wrapped with black tape and secured with a cable tie	Upper airway obstruction due to soil	NR	No	Oral cavity filled with soil/sand, with staining of the palate, tongue, and pharynx. Glottis occluded by a soil mass extending into the upper esophagus. Larynx, trachea, and main bronchi filled with impacted dark soil/sand	Lungs filled with impacted dark soil/sand	Marked cellular detail loss due to putrefaction	PMCT showed putrefactive changes and radio-opaque material filling the upper airway, consistent with soil. Blood toxicology negative for alcohol and common drugs
Halasi et al. [8]	2026	Case report	F/32	Severely ill woman affected by perforated duodenal ulcer and peritonitis, buried alive in a shallow grave inside a shed by her partner, who allegedly believed she was already dead	Mechanical asphyxia due to live burial, considered by airway obstruction from soil aspiration	Widespread soil contamination, especially on head and upper limbs; soil around mouth, ears and nose, scattered mid-facial hemorrhages, nasal deformity, multiple bruises and abrasions of different ages	No	Sandy/soil material present in the airways; soil particles detected deeply within trachea and lungs	Watery brain swelling with cerebellar tonsillar incarceration groove; swollen/overinflated lungs; chronic perforated duodenal ulcer, peritonitis and intra-abdominal fluid accumulation; bone scarring of left 9th–10th ribs	Acute emphysema; soil-derived particles identified in airways and lung tissue, including alveolar spaces, by polarized light microscopy	Toxicology negative for alcohol and drugs

NR = not reported; PMCT = Postmortem Computed Tomography.

**Table 2:** Methodological quality appraisal of the studies included in the review. The assessment was performed using eight predefined domains: demographics, history/timeline, clinical condition, diagnostic methods, intervention, post-intervention condition, adverse events, and takeaway lessons. The overall risk of bias was rated as low or moderate according to the completeness and reliability of the reported forensic and circumstantial information.

Study	Q1 Demographics	Q2 History/Timeline	Q3 Clinical Condition	Q4 Diagnostic Methods	Q5 Intervention	Q6 Post-Intervention Condition	Q7 Adverse Events	Q8 Takeaway Lessons	Overall Risk of Bias
Zarroug et al., 2004 [7]	Yes	Yes	Yes	Yes	Yes	Yes	Yes	Yes	Low
Benomran & Hassan, 2008 [5]	Yes	Yes	Yes	Yes	NA	NA	Yes	Yes	Low
Kiryu et al., 2018 [3]	Yes	Yes	Yes	Yes	NA	NA	Yes	Yes	Low
Kettner et al., 2008 [6]	Yes	Yes	Yes	Yes	Yes	Yes	Yes	Yes	Low
Byard, 2023 [1]	No	Yes	Yes	Yes	NA	NA	Yes	Yes	Moderate
Halasi et al., 2025 [8]	Yes	Yes	Yes	Yes	NA	NA	No	Yes	Moderate

NA = not applicable.

## Data Availability

The dataset generated during and analyzed during the current study are available from the corresponding author on reasonable request.

## Supplementary Materials

The following supporting information can be downloaded at: https://www.mdpi.com/article/10.3390/diagnostics16111691/s1, File S1: Full search strings; File S2: Risk of Bias.

## Abbreviations

CPR	Cardiopulmonary Resuscitation
GC–MS	Gas chromatography–mass spectrometry
H&E	Hematoxylin and Eosin (staining)
PMCT	Postmortem Computed Tomography
