# Supplementary material for: Asphyxial Mechanisms in Sand Burial, Findings and Diagnostic Challenges—A Case Report and a Literature Review
Source: Diagnostics (Basel). 2026 May 30;16(11):1691. doi: 10.3390/diagnostics16111691 (PMC13256983; doi:10.3390/diagnostics16111691)
Supplement: Supplementary file 1 [file diagnostics-16-01691-s001.zip › Supplementary Materials S1 28.05.2026.pdf]

Asphyxial mechanisms in sand burial, findings and diagnostic challenges – A case report and a literature review

Supplementary Materials S1

Full search strings

Pubmed
("bury"[Title/Abstract] OR "buried"[Title/Abstract] OR "burial"[Title/Abstract]) AND ("autop*"[Title/Abstract] OR "forens*"[Title/Abstract] OR "fatal*"[Title/Abstract]) AND (humans[Filter])

Initial findings: 565

Scopus
TITLE-ABS-KEY (bury OR buried OR burial) AND TITLE-ABS (autop* OR forens* OR fatal*) AND (LIMIT-TO (EXACTKEYWORD, "Human"))

Initial findings: 736
