# Supplementary material for: Asphyxial Mechanisms in Sand Burial, Findings and Diagnostic Challenges—A Case Report and a Literature Review
Source: Diagnostics (Basel). 2026 May 30;16(11):1691. doi: 10.3390/diagnostics16111691 (PMC13256983; doi:10.3390/diagnostics16111691)
Supplement: Supplementary file 1 [file diagnostics-16-01691-s001.zip › Supplementary Materials S2 28.05.2026.pdf]

Asphyxial mechanisms in sand burial, findings and diagnostic challenges – A case report and a literature review

Supplementary Materials S2

Risk of Bias

Risk of bias was assessed using JBI Critical Appraisal Checklist for Case Reports

"Accidental burials in sand: a potentially fatal summertime hazard". Zarroug et al., 2004 (1)

JBI Question	Rating	Supporting Information / Notes
**1. Were patient's demographic characteristics clearly described?**	Yes	Ages and sex were reported for all cases (boys aged 10–13 years). Weight was reported for the 2 fatal cases (40 kg and 45 kg). Previous health status described as "previously healthy". Ethnicity not reported.
**2. Was the patient's history clearly described and presented as a timeline?**	Yes	Circumstances leading to burial, estimated burial durations, rescue sequence, resuscitation efforts, transport, and outcomes were described chronologically for both fatal cases.
**3. Was the current clinical condition of the patient on presentation clearly described?**	Yes	Detailed presentation findings included:<br>- Case 1: unresponsive pupils, no pulse, no signs of life, no sand in mouth/pharynx/airway.<br>- Case 2: unresponsive, pulseless, no sand in airway, left hemotympanum, Battle sign, fixed dilated pupils, asystole, dependent mottling.
**4. Were diagnostic tests or methods and the results clearly described?**	Yes	Case 1: chest X-ray normal, no sand bronchogram. Case 2: no X-ray obtained. Clinical examination and resuscitation findings described. No autopsy findings were reported.
**5. Was the intervention(s) or treatment procedure(s) clearly described?**	Yes	CPR, airway management, intubation, transport, resuscitation attempts, and emergency response measures were described. Discussion section also details recommended management strategies for sand aspiration.
**6. Was the post-intervention clinical condition clearly described?**	Yes	After interventions, Case 1 had a weak pulse with sinus bradycardia and hypotension, then became asystolic and was pronounced dead. Case 2 remained asystolic with fixed dilated pupils and dependent mottling; resuscitation was discontinued.
**7. Were adverse events (harms) or unanticipated events identified and described?**	Yes	The unanticipated mechanism (death from thoracic compressive asphyxia rather than sand aspiration) is clearly identified and described, with calculations of sand weight (>3 tons). No unresolved diagnostic uncertainty.

JBI Question	Rating	Supporting Information / Notes
**8. Does the case report provide takeaway lessons?**	**Yes**	Accidental sand burial can cause death by compressive asphyxia without aspiration; weight of sand overwhelms respiratory muscles; prompt extrication and airway management are critical; public awareness and supervision needed.

“Accidental fatal asphyxiation by sand inhalation”. Benomran & Hassan, 2008 (2)

JBI Question	Rating	Supporting Information / Notes
**1. Were patient’s demographic characteristics clearly described?**	**Yes**	Age, occupation, expatriate worker status, sex, height, weight, and clothing were reported. Medical history was largely NR.
**2. Was the patient’s history clearly described and presented as a timeline?**	**Yes**	The report clearly described the sequence from workplace collapse, false witness account, hospital certification, forensic referral, radiology, autopsy, and subsequent police inquiry.
**3. Was the current clinical condition of the patient on presentation clearly described?**	**Yes**	The decedent was described as pronounced dead on arrival. Detailed external findings included extensive sand deposition in exposed orifices and absence of major trauma except nasal abrasion.
**4. Were diagnostic tests or methods and the results clearly described?**	**Yes**	Extensive forensic investigations were described, including external examination, radiography, full autopsy, histopathology, and toxicology. Radiographic and microscopic findings were clearly documented with figures.
**5. Was the intervention(s) or treatment procedure(s) clearly described?**	**NA**	This was a forensic postmortem investigation. No therapeutic intervention or treatment was performed prior to death aside from hospital pronouncement.
**6. Was the post-intervention clinical condition clearly described?**	**NA**	No clinical intervention/treatment course existed because the individual was dead on hospital arrival.
**7. Were adverse events (harms) or unanticipated events identified and described?**	**Yes**	The report described accidental occupational burial, fatal inhalation and swallowing of sand, diagnostic misclassification as myocardial infarction, and concealment of circumstances by coworkers.
**8. Does the case report provide takeaway lessons?**	**Yes**	Authors emphasized forensic vigilance, recognition of sand in exposed orifices, importance of medico-legal autopsy, and risks of relying solely on witness accounts.

“An autopsy report of accidental burial in a beach sand hole”. Kiryu et al., 2018 (3)

JBI Question	Rating	Supporting Information / Notes
**1. Were patient’s demographic characteristics clearly described?**	**Yes**	Ages, sex, marital status, height, weight, and limited toxicology information were reported for both decedents. Medical history was largely NR.
**2. Was the patient’s history clearly described and presented as a timeline?**	**Yes**	The report chronologically described excavation of the beach hole, covering with a plastic sheet, arrival of the couple, collapse event, rescue delay, transport, and pronouncement of death.
**3. Was the current clinical condition of the patient on presentation clearly described?**	**Yes**	The report clearly documented the condition at recovery and autopsy findings, including congestion, petechiae, pulmonary edema, and minimal sand aspiration.
**4. Were diagnostic tests or methods and the results clearly described?**	**Yes**	Detailed medico-legal autopsy findings, external/internal examinations, lung weights, toxicology, and scene reconstruction calculations were reported. Photographic figures supported findings.
**5. Was the intervention(s) or treatment procedure(s) clearly described?**	**NA**	This was a forensic postmortem investigation. No therapeutic intervention was described beyond rescue and transport.
**6. Was the post-intervention clinical condition clearly described?**	**NA**	No clinical treatment or post-intervention course existed because both victims were recovered deceased and pronounced dead at hospital.
**7. Were adverse events (harms) or unanticipated events identified and described?**	**Yes**	Fatal accidental burial, delayed rescue due to difficulty extracting victims, chest compression asphyxia, and potential neck compression were discussed extensively.
**8. Does the case report provide takeaway lessons?**	**Yes**	Authors emphasized the lethal risks of recreational beach-hole excavation, importance of public awareness, and dangers of chest compression even without significant sand aspiration.

“Fatal outcome of a sand aspiration”. Kettner et al., 2008 (4)

JBI Question	Rating	Supporting Information / Notes
**1. Were patient’s demographic characteristics clearly described?**	**Yes**	Age (2.5 years), sex (male), no past medical history, and context (sandbox) provided.
**2. Was the patient’s history clearly described and presented as a timeline?**	**Yes**	Chronology from playing to collapse, ambulance arrival (6 min), resuscitation, and death (45 min) clearly presented.
**3. Was the current clinical condition of the patient on presentation clearly described?**	**Yes**	Fixed pupils, no cardiopulmonary activity, sand in oral cavity/larynx, O2 saturation 60% pre-intubation and 80% post-intubation.
**4. Were diagnostic tests or methods and the results clearly described?**	**Yes**	Chest X-ray, autopsy, histology (emphysema), sand grain size analysis (0.35 mm), and photographs provided.
**5. Was the intervention(s) or treatment procedure(s) clearly described?**	**Yes**	Resuscitation efforts: sand removal from oral cavity/larynx, endotracheal intubation, epinephrine (3×1.5 ml 1:10,000), atropine (0.25 mg), sodium bicarbonate (15 ml), defibrillation (ascending doses 50, 100, 200 J).
**6. Was the post-intervention clinical condition clearly described?**	**Yes**	After interventions: spontaneous cardiopulmonary activity not reestablished, pupils remained fixed. Resuscitation terminated after 45 minutes.
**7. Were adverse events (harms) or unanticipated events identified and described?**	**Yes**	Unanticipated event (fatal self-administered sand aspiration in a child previously unreported) is identified and thoroughly described, including physical properties of dry sand and pathophysiology. No unresolved diagnostic uncertainty.
**8. Does the case report provide takeaway lessons?**	**Yes**	Fatal self-administered sand aspiration is possible in children; dry sand's physical properties worsen obstruction; emergency clinicians must suspect deep airway blockage.

"Homicidal burial - Forensic issues". Byard 2023 (5)

JBI Question	Rating	Supporting Information / Notes
**1. Were patient's demographic characteristics clearly described?**	**No**	Age ("young woman") and sex (female) are reported. Ethnicity, medical history, past treatments, and diagnostic history are not reported. The setting (shallow grave, sandy soil) is described. Due to respect for the family, detailed demographics were intentionally omitted.
**2. Was the patient's history clearly described and presented as a timeline?**	**Yes**	The decedent was abducted three days prior to exhumation. Death most likely occurred a few hours after kidnapping. No medical, family, or psychosocial history is provided, but these are not applicable to a homicidal burial case
**3. Was the current clinical condition of the patient on presentation clearly described?**	**Yes**	At discovery: buried sitting in shallow grave, hands tied behind back with cable tie, blindfolded, legs taped with black tape and cable tie. No significant injuries
**4. Were diagnostic tests or methods and the results clearly described?**	**Yes**	Postmortem CT showed radio-opaque material in upper airway and esophagus. Autopsy dissection confirmed soil/sand in mouth, pharynx, glottis, trachea, bronchi, and upper esophagus. Histopathology showed putrefactive changes. Toxicology was negative for alcohol and common drugs. Photographs are provided.
**5. Was the intervention(s) or treatment procedure(s) clearly described?**	**NA**	No clinical intervention or treatment was administered. This is a forensic autopsy case of a deceased individual.
**6. Was the post-intervention clinical condition clearly described?**	**NA**	No intervention was performed. The decedent was dead at discovery.
**7. Were adverse events (harms) or unanticipated events identified and described?**	**Yes**	The report identifies and describes multiple unanticipated forensic issues: determining if victim was alive at burial, level of awareness, mechanisms of death (smothering, choking, positional, crush asphyxia), and the effect of putrefaction slowed by burial (Casper's Law). No unresolved diagnostic uncertainty.
**8. Does the case report provide takeaway lessons?**	**Yes**	The discussion provides clear lessons: (1) homicidal burial is rare; (2) determining if victim was alive at burial requires autopsy evidence of soil inhalation; (3) mechanisms include smothering, choking, positional asphyxia, and crush asphyxia; (4) putrefaction complicates assessment but burial slows decay (Casper's Law); (5) restraints and absence of sedative drugs suggest the victim was conscious during burial.

“Severe illness as a risk factor for live burial”. Halasi et al., 2025 (6)

JBI Question	Rating	Supporting Information / Notes
**1. Were patient’s demographic characteristics clearly described?**	**Yes**	Age (32 years), sex (female), height (158 cm), and body habitus (well-developed, well-nourished) are reported. Medical history (perforated duodenal ulcer, peritonitis) is described. Ethnicity and previous treatments are not reported, but the essential medical context is provided.
**2. Was the patient’s history clearly described and presented as a timeline?**	**Yes**	The victim felt unwell for several days, went to bed feeling very weak, the perpetrator left to chop wood, returned to find her not moving, and then buried her in the shed. Police investigation determined she was abducted three days prior to exhumation in a related case reference, but the timeline here is less detailed than in clinical reports. Still, a sequential narrative is present.
**3. Was the current clinical condition of the patient on presentation clearly described?**	**Yes**	External examination is reported: at discovery, soil contamination on head/limbs, hypostasis, rigor mortis, soil around mouth/ears/nose, no fresh petechiae, old facial haemorrhages, bruises and abrasions of varying ages.
**4. Were diagnostic tests or methods and the results clearly described?**	**Yes**	Histopathology (acute emphysema), polarized light microscopy (soil particles in lung tissue), toxicology (negative for alcohol/drugs) and photographs are provided.
**5. Was the intervention(s) or treatment procedure(s) clearly described?**	**NA**	No emergency resuscitation or clinical intervention described. The victim was already dead when discovered by the perpetrator; no medical response occurred.
**6. Was the post-intervention clinical condition clearly described?**	**NA**	No intervention performed.
**7. Were adverse events (harms) or unanticipated events identified and described?**	**No**	The unanticipated event (live burial of a severely ill patient without resistance) is identified. However, the cause of death remains uncertain. The authors do not adequately address whether death resulted from airway obstruction by soil inhalation or from chest compression by the overlying soil. The absence of massive soil quantities in the airways leaves this differential diagnosis unresolved.
**8. Does the case report provide takeaway lessons?**	**Yes**	Severely ill individuals may be buried alive without resistance; soil in deep airways confirms respiration during burial; forensic pathology distinguishes live burial from postmortem concealment.

References

1. Zarroug AE, Stavlo PL, Kays GA, Rodeberg DA, Moir CR. Accidental Burials in Sand: A Potentially Fatal Summertime Hazard. Mayo Clinic Proceedings. 1 giugno 2004;79(6):774–6. doi:10.4065/79.6.774

2. Benomran FA, Hassan AI. Accidental fatal asphyxiation by sand inhalation. J Forensic Leg Med. agosto 2008;15(6):402–8. doi:10.1016/j.jflm.2008.01.008 PubMed PMID: 18586214.

3. Kiryu K, Takeichi T, Kitamura O. An autopsy report of accidental burial in a beach sand hole. Leg Med (Tokyo). novembre 2018;35:88–90. doi:10.1016/j.legalmed.2018.09.013 PubMed PMID: 30296738.

4. Kettner M, Ramsthaler F, Horlebein B, Schmidt PH. Fatal outcome of a sand aspiration. Int J Legal Med. novembre 2008;122(6):499–502. doi:10.1007/s00414-008-0252-4 PubMed PMID: 18546004.

5. Byard RW. Homicidal burial - Forensic issues. J Forensic Leg Med. novembre 2023;100:102617. doi:10.1016/j.jflm.2023.102617 PubMed PMID: 37950991.

6. Halasi BD, Borsay BÁ, Harsányi TG, Pórszász RK, Gergely PA. Severe illness as a risk factor for live burial. Forensic Sci Med Pathol. marzo 2026;22(1):324–31. doi:10.1007/s12024-025-01070-z PubMed PMID: 40802038; PubMed Central PMCID: PMC13132908.
